# Melatonin as a Mediator of the Gut Microbiota–Host Interaction: Implications for Health and Disease

**DOI:** 10.3390/antiox13010034

**Published:** 2023-12-23

**Authors:** María-Ángeles Bonmatí-Carrión, Maria-Angeles Rol

**Affiliations:** 1 Chronobiology Laboratory, Department of Physiology, College of Biology, Mare Nostrum Campus, University of Murcia, Instituto Universitario de Investigación en Envejecimiento, Instituto Murciano de Investigación Biosanitaria-Arrixaca, 30100 Murcia, Spain; angerol@um.es; 2 Ciber Fragilidad y Envejecimiento Saludable (CIBERFES), Instituto de Salud Carlos III, 28029 Madrid, Spain

**Keywords:** melatonin, gut microbiota, obesity, sleep, neurodegenerative disorders, metabolism, inflammatory bowel disease

## Abstract

In recent years, the role played by melatonin on the gut microbiota has gained increasingly greater attention. Additionally, the gut microbiota has been proposed as an alternative source of melatonin, suggesting that this antioxidant indoleamine could act as a sort of messenger between the gut microbiota and the host. This review analyses the available scientific literature about possible mechanisms involved in this mediating role, highlighting its antioxidant effects and influence on this interaction. In addition, we also review the available knowledge on the effects of melatonin on gut microbiota composition, as well as its ability to alleviate dysbiosis related to sleep deprivation or chronodisruptive conditions. The melatonin–gut microbiota relationship has also been discussed in terms of its role in the development of different disorders, from inflammatory or metabolic disorders to psychiatric and neurological conditions, also considering oxidative stress and the reactive oxygen species-scavenging properties of melatonin as the main factors mediating this relationship.

## 1. Introduction

Melatonin (N-acetyl-5-methoxytryptamine) is a pleiotropic molecule chemically classified as an indoleamine. It exerts multiple actions, both directly and through different receptors (MT1 and MT2, G-protein-coupled membrane receptors; MT3, cytosolic enzyme quinone reductase 2, which has potent antioxidant effects when bound to melatonin). In mammals, this hormone is produced by the pineal gland, located in the brain, as well as by a great variety of extrapineal sources, including the retina and the immune and gastrointestinal systems (reviewed in [1]).

**Figure 1:**
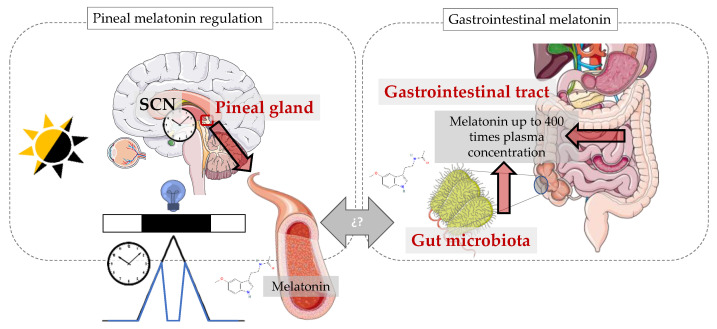
Pineal and gastrointestinal melatonin regulation. This figure was built with SMART resources (Servier Medical Art), licensed under a Creative Common Attribution 3.0 Generic License. See http://smart.servier.com/ (accessed on 1 September 2023).

Among the functional properties of melatonin, we can find potent antioxidant (to be reviewed in the next section), immunomodulatory and anti-inflammatory actions, as well as specific antiproliferative, antiangiogenic and proapoptotic effects in tumour cells (reviewed in [1]). Apart from these local effects, melatonin is also considered to be the most important chronobiotic hormone when released by the pineal gland into the blood circulation, sending time-of-day information and acting as a chemical signal of darkness (reviewed in [2]). This hormone presents an endogenous circadian variation, with a peak during the night and undetectable levels during the day. In addition, pineal melatonin can be acutely suppressed by light at night. Both processes are essential for this molecule to inform about the arrival of darkness at night. Although melatonin locally produced in other tissues has also shown different circadian patterns, the interplay between the pineal and extrapineal melatonin rhythms is not yet clear (reviewed in [1]) (Figure 1). 

The most important extrapineal source of melatonin is precisely the gastrointestinal tract, where the concentrations can reach levels as much as 400 times greater than plasma melatonin levels [3]. Also, a healthy gut contains an enormous population of microorganisms working in close relationship with our body and physiology. Traditionally, the gut microbiota has been known to be involved in metabolic activities that result in energy and nutrient management and protection of the host against invasion by pathogenic microorganisms. However, in recent years, the gut microbiota has been shown to be involved in a myriad of functions. Indeed, dysbiosis (abnormal gut bacterial composition) has been linked to the pathogenesis of several inflammatory disorders, as well as to the emergence of certain neurological conditions and even cancer. It is now well known that changes in our physiology can affect the gut microbiota and, conversely, that changes in the gut microbiota will affect our physiology. So, knowing the particular processes involved in this interplay will be essential in order to treat and prevent a number of health issues.

In this sense, maintaining a healthy microbiome composition becomes crucial to preserving health and wellbeing. Although nutrition is thought to be the primary factor affecting the gut microbiota, other aspects may also compromise the balance between commensal and pathogenic gut bacteria. It has been shown that a lack of sleep alters melatonin levels in the gut [4] and plasma [5], as well as the gut microbiota composition [4,5,6,7,8,9,10]. It is precisely in the relationship between the gut microbiota, sleep and circadian rhythms where melatonin may play an important role in diurnal species, such as humans. Indeed, sleep deprivation and artificial light at night [11] have been demonstrated to suppress melatonin and to alter the microbiota composition, an effect that can be counteracted via the administration of exogenous melatonin [12].

Considering that melatonin has been identified in different and distant taxa of unicellular organisms, including bacteria [13], it is not surprising to find that the gut microbiota is also a source of melatonin [14] and contains melatonin receptors. This means that melatonin might also act as a signal to transfer both external and internal information between the gut microbiota and the host.

Therefore, melatonin may play a significant role in the relationship between sleep deprivation or artificial light at night, dysbiosis and those health problems that may result from alterations of the microbiota composition. It would follow that among other multiple functions of melatonin, maintenance of the microbiota composition could be another crucial role of this pleiotropic molecule.

All of this suggests that the gut microbiota might be acting through melatonin as a link between two essential aspects of health: nutrition and sleep, both framed within the circadian system. Here, we will review the current scientific evidence on the relationship between melatonin and the gut microbiota, as well as the effects of that interplay on the development of different health issues, excluding cancer, which has already been extensively reviewed in [1].

## 2. Antioxidant Properties of Melatonin

Maintaining a balance between the production and removal of free radicals is crucial for overall health, as an imbalance leads to oxidative stress. This condition results in macromolecular damage, potentially affecting the integrity of the gut barrier. Melatonin, the original function of which in unicellular organisms is speculated to be as an antioxidant, plays a protective role against oxidative stress at molecular, cellular, tissue, organ and organ system levels [15] through direct free radical scavenging and indirect modulation of antioxidant enzyme expression. Additionally, melatonin has the ability to repair oxidised biomolecules [16].

Antioxidants can be categorised based on their chemical mechanisms of action against oxidative stress. Melatonin has been proposed to be a Type IV or multifunctional antioxidant. Also, as a Type I antioxidant, melatonin can detoxify various reactive oxygen species (ROS) and reactive nitrogen species (RNS) by directly reacting with them or through the generation of less reactive species. The indole moiety of melatonin plays a crucial role in its antioxidant capacity, as it interacts with free radicals due to its resonance stability. Melatonin can scavenge multiple reactive species through a “free radical scavenging cascade”, making it a highly effective antioxidant [17]. This cascade starts once melatonin interacts with reactive species, generating intermediaries that are, in turn, free radical scavengers with different efficiencies and specificities (reviewed in [18]). As a result of this mechanism, melatonin becomes a very effective antioxidant [19,20]. These metabolites include [18] N-acetylserotonin, 5-methoxytryptamine, cyclic 3-hydroxymelatonin, N^1^-acetyl-N^2^-formyl-5-methoxykynuramine, N^1^-acetyl-5-methoxykynuramine, 6-hydroxymelatonin, 4-hydroxymelatonin and 2-hydroxy melatonin.

Melatonin also acts as a Type II antioxidant by quenching singlet oxygen (^1^O_2_) [21,22], chelating metal ions [23] and inhibiting lipid peroxidation and the protein damage induced by metal interactions [24,25,26]. Additionally, as a Type III antioxidant, melatonin regenerates other antioxidants, such as glutathione, ascorbic acid and Trolox, through electron transfer processes [27,28]. Melatonin can repair oxidised DNA via electron transfer [29,30] and also by enhancing DNA repair mechanisms [31,32,33,34], activating antioxidant enzymes [35,36,37,38,39,40,41] and inhibiting pro-oxidative enzymes in non-tumour cells [42,43]. 

These unique antioxidant properties of melatonin, including the free radical-scavenging cascade, place it as a central molecule in terms of antioxidant activity. Considering the concentrations required for these purposes, it is important to note that it is extrapineal melatonin, synthesised outside the pineal gland, which plays a significant role in antioxidant and anti-inflammatory processes. However, we cannot ignore the potential relevance of the interplay between pineal and extrapineal melatonin, which remains an important area of research to better understand the regulation of the antioxidant functions of melatonin.

## 3. ROS in the Gut

Reactive oxygen species (ROS) can be beneficial in low and moderate concentrations. However, when ROS levels increase, they can have detrimental effects, and their impact varies depending on the concentration and the specific tissues involved. ROS play a role in the health of the intestine and the diversity of the gut microbiota (reviewed in [44]). Maintaining the health of this organ relies to an important extent on the intestine’s ability to effectively regulate the excessive production of ROS. Indeed, the balance of gut microbiota composition is closely and bidirectionally associated with the redox equilibrium. In this sense, oxidative stress has been demonstrated to alter the gut microbiota composition in mice, increasing *Bacteroidetes* and decreasing *Firmicutes*, *Clostridiales*, *Ruminococcaceae* and *Oscillospira* [45]. On the other hand, the presence of gut injury and dysbiosis can lead to the suppression of antioxidant activity in the gastrointestinal tract [46]. Under such circumstances, melatonin can exert its antioxidant activity, restoring the redox balance and thereby enhancing the composition of the gut microbiota, as will be detailed in the following sections. 

The regulation of the gut microbiome and systemic oxidative stress is interconnected and complex. Oxidative stress occurring in the gut can result in damage to the structural integrity of the intestinal barrier. Specifically, damage to mitochondrial DNA in intestinal epithelial cells can lead to excessive local production of ROS, contributing to mitochondrial dysfunction. This further exacerbates the increase in ROS levels and oxidative damage and, in addition, leads to a decrease in the expression of tight junction proteins (Occludin, Claudin-1 and ZO-1) and the death of intestinal epithelial cells [47].

Indeed, an important factor in the pathogenesis of gut microbes is bacterial translocation due to the compromised integrity of the intestinal barrier. The lipopolysaccharide (LPS) response serves as a significant marker signal in this process, which can activate abnormal immune signals by binding to the TLR-4 complex, subsequently increasing the production of ROS/RNS and proinflammatory cytokines [48,49]. Also, LPS can stimulate the expression of oxidative stress-related enzymes, such as inducible nitric oxide synthase [50] or NADPH oxidase, resulting in excessive RNS/ROS production and activation of the downstream NF-κB pathway [51,52]. Furthermore, specific gut microbes can induce the production of ROS in intestinal epithelial cells, and it has been shown that *Lactobacillus* can induce the production of ROS by intestinal phagocytes depending on the presence of NADPH oxidase 1 [53].

ROS levels in the gut are counteracted by the antioxidant activity of gut microbes, facilitated by short-chain fatty acids (SCFAs), among other metabolites. SCFAs inhibit peroxisomes and activate the Nrf2 pathway, contributing to the maintenance of redox balance in the gut [54]. Thus, gut microbiota play a crucial role in regulating this balance, which is essential for gut health and overall wellbeing.

### Gut Melatonin and ROS

Melatonin acts in the gut not only by scavenging highly toxic ROS but also by upregulating different antioxidant enzymes, including glutathione peroxidase, catalase and superoxide dismutase. Additionally, melatonin downregulates pro-oxidative enzymes, further contributing to the maintenance of a favourable redox environment in the gut. Melatonin can exert its effects through direct interactions with ROS, as well as via membrane and nuclear receptors that act as mediators for its indirect antioxidant actions. Through this mechanism, melatonin activates various stress-responsive genes, such as Sirt, HIFa and AMPK, triggering an increase in the expression of multiple antioxidant enzymes [55]. Consequently, the significant impact of melatonin on the enteric microenvironment stems largely from the reduction in oxidative stress exerted on the gut microbiota through the various pathways mediated by melatonin.

As examples that confirm this evidence, exposure to imidacloprid (a neonicotinoid insecticide) has been shown to induce gut toxicity in zebrafish via oxidative stress, concomitantly with a decrease in melatonin and serotonin levels. In this study, the prolonged darkness that increased melatonin levels also attenuated said toxicity [56]. Melatonin administration can also ameliorate skin oxidative stress indirectly through the regulation of propionic acid as observed in a model of sleep-restricted mice [57]. Also in sleep-restricted mice, melatonin has been shown to prevent intestinal dysbiosis by palliating oxidative stress [5,6,8,9]. In mice with DSS-induced colitis, melatonin has also been demonstrated to improve oxidative stress resistance while regulating the gut microbiota and improving overall intestinal health [58].

## 4. Melatonin Effects on Gut Microbiota Composition

The gastrointestinal tract has an intimate relation with gut microbiota, and it is well known that keeping this dynamic population of microorganisms in good shape contributes to maintaining immune status and metabolic homeostasis. The gut microbiota also helps to defend the host against pathogens, reducing the probability of infections through the intestinal barrier. In this sense, and as reviewed below, both host and exogenous melatonin have been shown to have an impact on the gut microbiota, producing compositional, metabolic and circadian changes. 

Two characteristics of the microbial gut microbiota are (i) its abundance (more than 100 trillion microorganisms, 10^11^–10^12^ per millilitre) and (ii) its diversity. To date, the Human Microbiome Project and MetaHit data have presented the most complete picture of the human-associated microbial repertoire [59,60]. These studies made it possible to identify 2172 human-isolated species that belong to 12 phyla, with 93.5% corresponding to *Proteobacteria*, *Firmicutes*, *Actinobacteria* and *Bacteroidetes*. One of these publications [59] also included a comprehensive inventory of the functional potential of the human gut microbiome, finding 9,879,896 genes that present a high level of redundancy. 

Despite the high degree of redundancy found in gut microbial gene expression, an imbalance in this community can lead to health impairment. This alteration in microbiota composition is known as “dysbiosis” and has been linked to a variety of diseases, such as inflammatory bowel disease (IBD), metabolic disorders (e.g., obesity or diabetes), allergies, cancer and neurologic conditions, such as autism spectrum disorders (reviewed in [61]). Given the importance of maintaining the gut microbiota composition and preventing/reverting dysbiosis, the factors involved in these processes have recently been gaining attention.

➢ Effects of exogenous melatonin

Although the exact mechanism has not yet been unravelled, exogenous melatonin administration causes different changes in gut microbiota composition (see Table 1). Indeed, melatonin has been shown to reduce the *Firmicutes*/*Bacteroidetes* ratio (which is considered an indicator of intestinal homeostasis; decreased *Firmicutes*/*Bacteroidetes* ratios are associated with dysbiosis) and also to increase the abundance of *Akkermansia*, a mucin-degrading bacteria related to a healthy mucosa. Exogenous melatonin has been demonstrated to revert 14 of the 69 operational taxonomic units (OTUs) altered in mice fed a high-fat diet, including *Desulfovibrionaceae, Bacteroides, Ruminococcaceae*, *Helicobacteraceae, Porphyromonadaceae* and *Christensenellaceae* [62]. Other authors found that melatonin had the effect of increasing *Clostridiales*, *Bacteroidales* and *Enterobacteriales* [63], *Alistipes* [64] and *Bacteroidetes* [65] and decreasing *Firmicutes* in rats [63] and mice [64,65] fed a high-fat diet.

Ren and colleagues (2018) [12] explored the effects of melatonin supplementation in mice suffering from weanling stress, finding that 0.2 mg/mL for 2 weeks was enough to influence the gut microbiota composition and to increase its richness indices, with an increase in the abundance of *Lactobacillus*, while reducing the load of enterotoxigenic *Escherichia coli* [12,66]. Similar effects were reported by Jing and colleagues in 2019, who demonstrated an increase in the relative abundance of *Lactobacillales* and *Lactobacillus* after injecting melatonin in a mouse model of spinal cord injury suffering from the consequent dysbiosis. In this case, the relative abundance of *Clostridiales* was reduced by the melatonin treatment [67]. Other studies that found a protective effect of melatonin in colitis symptoms discovered that although its administration reduced the variety and richness of the gut microbiota, it also increased the amount of the probiotic *Bifidobacterium* and at the same time decreased the relative abundance of pathogenic bacterial taxa, such as *Desulfovibrio*, *Peptococcaceae* and *Lachnospiraceae* [68]. In suckling piglets, melatonin has been shown to increase the relative abundance of *Actinobacteria* while reducing *Selenomonadales* [69].

**Table 1:** Effects of melatonin on gut microbiota composition.

Study	Animal Model	Treatment to Alter Gut Microbiota Composition	Melatonin Treatment/Intervention (Route, Dosage, Time Period)/ Measurement	Effects of Melatonin Treatment
Xu et al., 2017 [62]	Mouse	High-fat diet	By gavage 50 mg/kg body weight Once daily, 10 weeks	Revert 14/69 OTUs altered ↓ *Firmicutes*/*Bacteroidetes* ↑ *Akkermansia*
Yildirim et al., 2019 [63]	Rat	High-fat diet	In drinking water 4 mg/kg per day 2 weeks	↑ *Clostridiales* ↑ *Bacteroidales* ↑ *Enterobacteriales*
Yin et al., 2018 [64]	Mouse	High-fat diet	In drinking water 0.4 mL/mL 2 weeks	↑ *Bacteroides* ↑ *Alistipes*
Ren et al., 2018 [12]	Mouse	Weanling mouse model	In drinking water 0.2 mg/mL 2 weeks	↑ Richness ↑ *Lactobacillus* ↓ *Escherichia coli*
Jing et al., 2019 [67]	Mouse	Mouse model of spinal cord injury	Intraperitoneal injection 10 mg/kg Twice a day	↑ *Lactobacillales* ↑ *Lactobacillus* ↓ *Clostridiales*
Li et al., 2021 [70]	Sheep	*Brucella* infection	Overexpression of ASMT in transgenic sheep	↓ Abundance of microbes related to infectious diseases
Zhang et al., 2022 [71]	Mouse	-	Endogenous melatonin reduction (Aanat-knockout (Aanat−/−))	Microbiota dysbiosis ↑ Gut permeability ↑ Systemic inflammation
Zhao et al., 2022 [68]	Mouse	Induced colitis (oxazolone)	50 mg/kg body weight By gavage 1 week before induction of colitis	↑ *Bifidobacterium* ↓ *Desulfovibrio* ↓ *Peptococcaceae* ↓ *Lachnospiraceae*
Xia et al., 2022 [69]	Sucking piglets	Healthy	10 mL oral melatonin solution (1 mg/mL) 21 days	↑ *Actinobacteria* ↓ *Selenomonadales*
Ouyang et al., 2021 [72]	Lactating cows	Healthy	Ruminal melatonin In vitro	(+) *Muribaculaceae,* *Succinivibrionaceae, Rikenellaceae,* *unidentified Cyanobacteria, Defluviitaleaceae, Veillonellaceae, Spirochaetaceae* and *Prevotellaceae* ↑ *Prevotellaceae* ↑ *Muribaculaceae* ↓ *Succinivibrionaceae* ↓ *Veillonellaceae*
Yin et al., 2020 [65]	Mouse	High-fat diet		↑ *Bacteroidetes* ↓ *Firmicutes*

➢ Manipulated endogenous melatonin production

**Figure 2:**
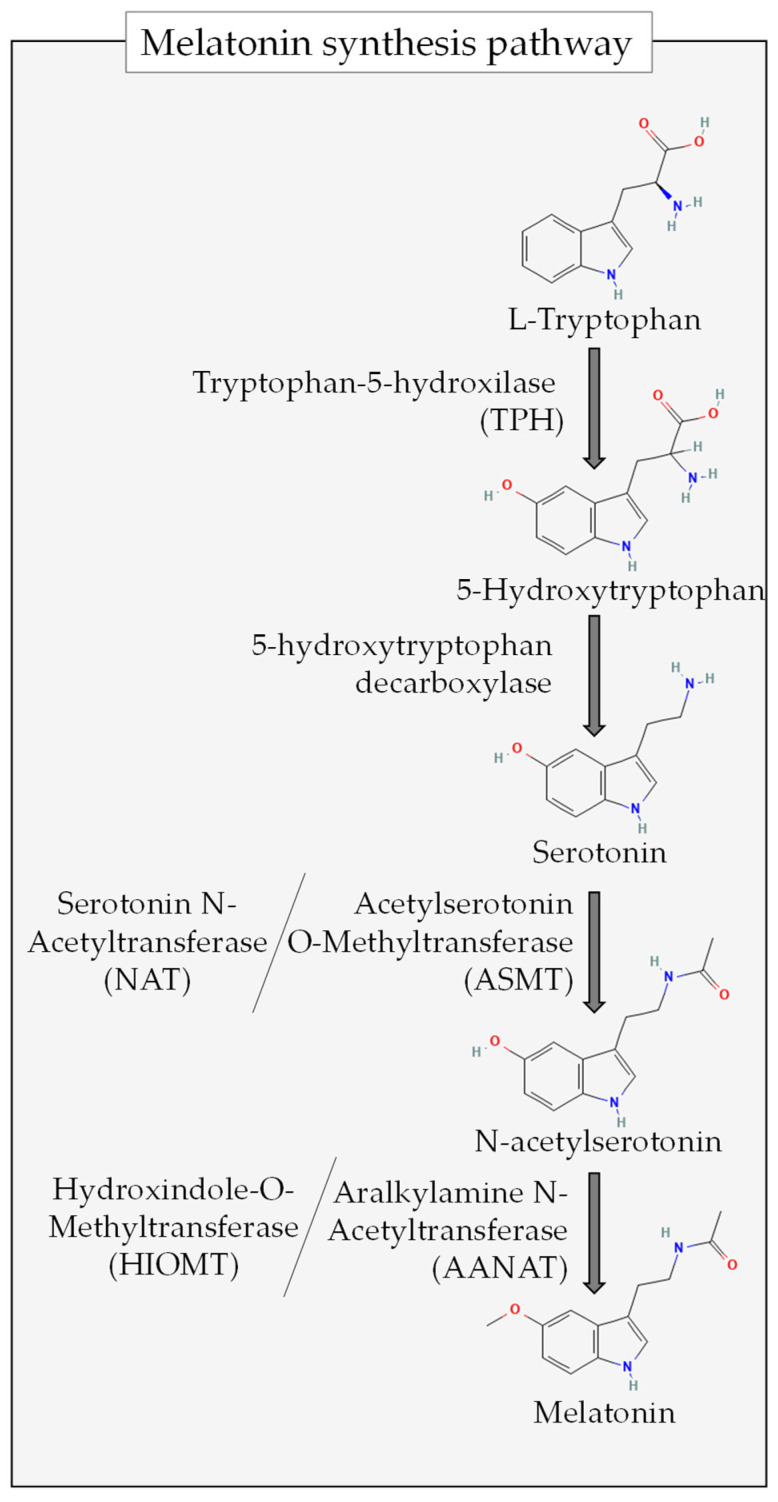
Melatonin synthesis pathway. Arrows mean the direction of the synthesis pathway.

Another approach to studying the possible effects of melatonin on gut microbiota composition is through the use of transgenic animals over- or underexpressing limiting enzymes for the synthesis of melatonin (e.g., aASMT, Figure 2). The overexpression of these enzymes has been shown to reduce the abundance of microbes related to infectious diseases in transgenic sheep [70]. In contrast, the models with endogenous melatonin reduction (EMR) suffer from microbiota dysbiosis, together with systemic inflammation and increased gut permeability [71].

## 5. Effects of Melatonin on Rhythmic Variations of Microbiota

The gut microbiota imbalance, known as dysbiosis, should not be confused with the normal variation of gut microbiota composition, considering that this microbial population is not an isolated and static bacterial community. Instead, its composition and metabolism depend on different factors. Some of these factors are exogenous, such as diet; some are endogenous, such as host genetics; and some have an exogenous component mediated by endogenous physiological processes. An example of the latter is the light–dark cycle or photoperiod, the effects of which are mediated by melatonin secreted in a circadian manner by the pineal gland. Thus, the gut microbiota shows a daily metabolic rhythmicity, partly influenced by the host circadian system, which is also affected by the rhythmic variations of microbiota in a bidirectional way. The physiology of a healthy gut microbiota includes this rhythmicity, with daily variations in community populations as well as circadian variations in their functional and metabolite expression [73]. 

Although the evidence so far is limited, some results support the potential beneficial effect of melatonin supplementation in restoring the lost rhythmicity of *Firmicutes* under a high-fat diet [65], for example. This study includes a detailed analysis of the rhythmicity exhibited by different phyla and genera under an HFD and melatonin supplementation, with varying results.

Another interesting area of study is endogenous melatonin of extrapineal origin. Although the information is limited, the role of melatonin produced by the gastrointestinal tract (which can have a concentration of up to 400 times that of melatonin from pineal origin) has been investigated in ruminants by Ouyang et al. (2021). They found that melatonin in the rumen fluid oscillates in a diurnal manner and so does the relative abundance of 9% of total rumen bacterial OTUs. Furthermore, ruminal melatonin seems to be positively correlated with *Prevotellaceae*, *Muribaculaceae*, *Veillonellaceae* and *Succinivibrionaceae*. Apart from this in vivo experiment, these authors also confirmed the effect of melatonin on bacterial composition with an in vitro assay, finding that melatonin can increase the relative abundance of *Muribaculaceae* and *Prevotellaceae* while reducing that of *Veillonellaceae* and *Succinivibrionaceae*. According to these results, ruminant microbiota seems to maintain a circadian rhythm associated with melatonin profiles [72].

## 6. Effects of Melatonin on Other Aspects of Gut Microbiota

Composition or rhythmicity are not the only aspects of the gut microbiota to be affected by melatonin. In fact, in vitro swarming and motility of *Enterobacter aerogenes* can be influenced by melatonin (1 nM), which also produces a synchronising effect of this rhythmic behaviour. This effect might be mediated by the presence of melatonin receptors found in this prokaryote [74]. Although *Klebsiella pneumoniae* and *Escherichia coli* do not seem to be sensitive to the effect of this indoleamine [74], the latter has also shown a dose-dependent repellent response to melatonin (<0.1 mM) [66].

Short-chain fatty acids (SCFAs), the main metabolites produced by the gut microbiota, exert many different and relevant functions in our body. They are produced by anaerobic fermentation of indigestible polysaccharides (e.g., dietary fibre or resistant starch) [75], and their presence in plasma also exhibits a diurnal rhythm that is disrupted under chronodisruptive conditions, such as night shift work [76]. 

## 7. Gut Microbiota as an Extrapineal Source of Melatonin

Some of the demonstrated effects of melatonin on the gut microbiota have been described in previous sections. However, the relationship between melatonin and the gut microbiota has been shown to be bidirectional, so this population of microbes can also affect melatonin levels in the host. Indeed, since microorganisms can produce large amounts of melatonin [13,77], intuitively, we may infer that the intestinal microbiota could be another source of melatonin [14].

One approach to demonstrating the ability of the gut microbiota to produce melatonin has consisted of injecting a precursor of the indoleamine (5-Hydroxytryptophan, 5-HTP) into the gastrointestinal tract and evaluating whether melatonin increases at different levels (see Figure 2 for melatonin synthesis pathway). Pan et al. (2021) identified the highest concentrations of the hormone in the colon, which suggested that the microbiota present at that level of the gastrointestinal tract may participate in melatonin production. These authors also demonstrated that melatonin produced in the gut can also reach the bloodstream via the portal vein, since sheep injected with 5-HTP also exhibited higher plasma melatonin concentrations [78]. However, considering that the possible interplay between melatonin from pineal and extrapineal sources has not yet been elucidated, this statement might be considered questionable.

Another indirect method for detecting possible actions of the gut microbiota on the host’s melatonin levels is through the effects of microbial metabolites. In this sense, butyrate (an SCFA produced by gut microbiota) has been shown to induce N-acetylserotonin and melatonin synthesis in the gut through the induction of aralkylamine N-acetyltransferase (AANAT) and acetylserotonin O-methyltransferase (HIOMT) (two limiting enzymes for melatonin production; see Figure 2), suggesting that a possible effect of this SCFA might be mediated via its induction of the melatonergic pathway, not only in the microbiota itself but also in (e.g.,) immune cells [79]. Also, the induction of this indoleamine through butyrate may exert feedback on the gut microbiota through the mechanisms described in Section 3.

Another way in which microbiota might influence the effects of melatonin on the host is by inducing the expression of melatonin receptors. In this sense, Wang et al. demonstrated in 2019 that gut microbiota is able to promote melatonin receptor expression in the host’s colonic cells, and they suggested that SCFAs may be relevant actors in the mechanism involved [80].

## 8. Sleep, Melatonin and Microbiota

There is increasing evidence that suggests that sleep deprivation produces gut microbiota disorders and that melatonin might be a possible mediator between the two processes [4,5,6,7,8,9,10] (Table 2).

In 2019, Gao et al. [6] demonstrated that 72 h sleep deprivation can produce colitis in mice, with a reduction in the diversity and richness of the gut microbiota, especially decreasing probiotics such as *Akkermansia*, *Bacteroides* and *Faecalibacterium* while increasing the pathogen *Aeromonas*. Concomitant with these changes, an increase in nor-epinephrine and a reduction in plasma melatonin were detected, with the consequent reduction in antioxidant ability and down- and upregulation of anti-inflammatory and proinflammatory cytokines, respectively, which led to colonic mucosal injury. Supplementation with 20 and 40 mg/kg of melatonin was able to reverse these changes, palliating mucosal injury and dysbiosis.

These authors [5] also reported that melatonin supplementation increased plasma melatonin concentration and OTUs, improving the diversity and richness of jejunal microbiota by increasing the presence of *Bacteroidaceae*, *Prevotellaceae*, *Moraxellaceae* and *Aeromonadaceae* and increasing the *Firmicutes*/*Bacteroidetes* ratio. Melatonin also increased anti-inflammatory cytokines (IL-22), decreasing proinflammatory cytokines (IL-17) and ROS, which resulted in a reduction in inflammation and oxidative stress. Similarly, a protocol of 20 h of sleep deprivation during 48 days in mice produced a reduction in plasma melatonin (48.91%), antioxidant enzymes (and total antioxidant capacity in intestinal tissues) and anti-inflammatory cytokines (IL10 and IFNγ). This protocol also produced an increase in glucose, norepinephrine, corticosterone and proinflammatory cytokines (IL6 and TNFα). However, it also increased α-diversity by increasing *Helicobacter* and *Clostridium* and reducing *Bacteroidetes* and *Lactobacillus*. Again, melatonin supplementation was able to restore these phenotypes [9]. These authors also described the possible role of corticosterone, which is increased in sleep-deprived mice, in mediating the colitogenic effect of sleep deprivation through microbiota. Indeed, the beneficial effects of melatonin might be related to its weakening action on glucocorticoid receptor feedback through the MT2 receptor [8].

These authors went a step further and found that sleep-deprived mice showed gut microbiota disorders and mucosa injuries, together with a decrease in plasma melatonin concentration, Card9 expression and *Faecalibacterium*, with the consequent reduction in butyrate levels. Melatonin supplementation, however, reversed all of these effects produced by sleep deprivation. The fact that the colitis induced in healthy (non-sleep-deprived) mice via faecal transplantation from sleep-deprived animals was reverted through the supplementation of butyrate, with no change in melatonin concentration, demonstrates that melatonin should be acting in mucosal integrity through the restoration of the microbiota [7].

The role of the interactions between *Aeromonas* and goblet cells has been also explored in mice, finding that *Aeromonas veronii* and LPS supplementation (naturally increased in sleep-deprived animals) can mimic the reduced number of goblet cells and mucin protein produced during sleep deprivation, while melatonin is capable of reversing these effects, probably through MT2 receptors [10].

Park and colleagues [4] also demonstrated the interplay between sleep deprivation, melatonin and microbiota in mice, revealing a decrease in melatonin levels—this time measured in faeces and the colon—concomitant with a shift to a colitogenic microbiota (increasing *Erysipelotrichales* and *Enterobacteriales* and decreasing *Lactobacillales*). This microbiota shift was reverted through melatonin supplementation, with an increased presence of *Akkermansia muciniphila* and *Lactobacillus*, while *Bacteroides massiliensis* and *Erysipelotrichaceae* were reduced.

**Figure 3:**
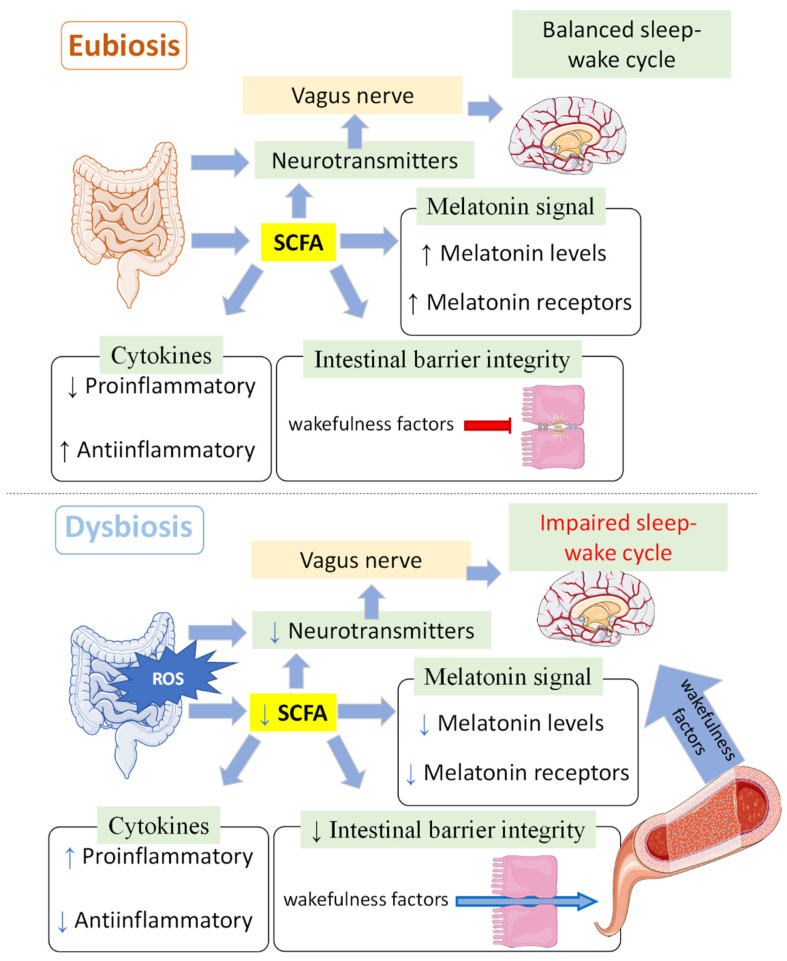
Mechanisms involved in the relationship between dysbiosis and an impaired sleep–wake cycle. SCFAs: short-chain fatty acids. This figure was created with SMART resources (Servier Medical Art), licensed under a Creative Common Attribution 3.0 Generic License. See http://smart.servier.com/ (accessed on 1 September 2023).

However, the exact mechanism involved in the relationship between microbiota and sleep homeostasis and the role of melatonin has yet to be determined (Figure 3). Considering that most of the sleep deprivation protocols produce an impairment of antioxidant ability, an effect that can be reverted via melatonin administration, the antioxidant properties of this molecule cannot be excluded from this possible mechanism. Indeed, various studies have examined the beneficial impact of administering melatonin on oxidative status following the implementation of diverse sleep deprivation protocols [5,6,9].

In this sense, most of the physiological conditions and pathologies leading to poor sleep quality have been related to enhanced oxidative stress. Sleep deprivation has been demonstrated to increase oxidative stress either by increasing ROS generation or by decreasing antioxidant capacity [81,82,83,84,85]. Not only deprivation but also sleep fragmentation can be responsible for an increase in oxidative stress [86]. Pathologies like insomnia [87,88] or conditions like circadian disruption [89], a widely accepted cause for poor sleep quality, have been related to higher levels of oxidative stress. Therefore, and considering diurnal animals such as humans (where melatonin serves not only as a chronobiotic but also as a sleep inducer [90,91,92]), it becomes plausible that the potential positive impact of melatonin on sleep quality [93] could extend to improvements in microbiota status. This amelioration may be mediated by the enhancement of oxidative status, suggesting a multifaceted role for melatonin in promoting overall health.

On the other hand, healthy microbiota will maintain the production of neurotransmitters, both directly and indirectly, through SCFA production, which will activate enterochromaffin cells, which in turn secrete different neurotransmitters [94]. However, most neurotransmitters cannot pass the intestinal and the blood–brain barriers [95], so those molecules produced in the intestine will act through the vagus nerve, which contains receptors and can send the signal to the brain [96]. The melatonin signal will also be enhanced not only by direct melatonin production but also through the upregulation of its receptors mediated by SCFAs [80]. SCFAs are also immunomodulators that reduce proinflammatory cytokines and increase anti-inflammatory cytokines [75,97,98,99], which, together with the better union between epithelial cells [98,100,101], will prevent different wakefulness factors from being included in systemic circulation. When the gut microbiota composition/homeostasis is disrupted (dysbiosis), the reduction in SCFAs, together with the increase in lipopolysaccharides, will produce an increase in proinflammatory cytokines and will make both the blood–brain barrier and the intestinal barrier more permeable, favouring the passage of these molecules to systemic circulation [101], activating the hypothalamic–pituitary–adrenal axis and producing wakefulness neurotransmitters/hormones (e.g., norepinephrine, cortisol) [96,101,102]. 

At this point, it should be mentioned that most of the publications linking sleep deprivation, melatonin suppression and gut microbiota disorders have focused on mice, a nocturnal animal model, so it could be of interest to extrapolate these results to humans as diurnal animals. 

To the best of our knowledge, there is not much research on humans that directly evaluates sleep, melatonin and microbiota composition in a polyhedral manner; however, there are some studies that attribute sleep benefits to the consumption of pro- (live microorganisms), pre- (foods that promote the growth of beneficial bacteria in the gut) and postbiotics (bioactive compounds resulting from the digestion of prebiotics by gut microbiota). Although there is some controversy, most of these studies show that the consumption of pre-, pro- and postbiotics improves sleep quality in humans (extensively reviewed in [103]).

## 9. Circadian System, Melatonin and Gut Microbiota

Sleep is regulated through two different types of processes, homeostatic and circadian, and it is not in vain that the sleep–wake cycle is considered to be one of the major outputs of the circadian system, also acting as a *zeitgeber* (*zeitnemer,* since it can act as a circadian input and output). Therefore, the circadian system must be highly involved in the interplay between sleep, melatonin and gut microbiota. Firstly, SCFAs show day/night fluctuations in mice, with increased levels during the active periods [104]. Furthermore, although the literature is limited, SCFAs have been demonstrated to synchronise *Per2/Bmal1* (two core pieces of the molecular clock) in hepatocytes [105] and to produce phase advance in all clock genes in the kidney and in *Per2* and *Bmal1* in submandibular gland cells when administered in the middle of the day [104], also inhibiting histone deacetylation [75]. Therefore, SCFAs, the main metabolites of gut microbiota, could act as a signal on the circadian clock.

Unconjugated and secondary bile acids have been shown to promote circadian rhythmicity by increasing *Per* and *Cry* expression in the ileum and colon [106,107]. Interestingly, the bacteria present in healthy gut microbiota (e.g., *Lactobacillus*, *Bifidobacterium*, *Clostridium* and *Enterococcus*) produce bile salt hydrolase (BSH), which in turn will deconjugate bile acids, allowing them to exert their beneficial effects on the circadian system [106]. Sleep disruption, on the other hand, will reduce the presence of *Lactobacillus* and *Bifidobacterium* and, therefore, the production of BSH [108].

➢ Influence of circadian cues on microbiota composition and effects on (and of) melatonin

Although it would require a more complex experimental design and the conclusions are rather speculative, some authors have tried to assess the possible effects of endogenous melatonin through the manipulation of environmental conditions (Table 3).

The light/dark cycle, produced by the alternation of day and night driven by the Earth’s rotational movement, is considered to be the main input of the circadian system. Melatonin produced by the pineal gland—mainly at night and in darkness—is closely tied to this cycle, presenting an endogenous rhythm driven by the suprachiasmatic nuclei (central pacemaker), which is also synchronised, thanks to this cycle. Apart from this endogenous rhythmicity, melatonin secretion is also acutely suppressed by light at night (Figure 1). The sleep-promoting effects of melatonin are restricted to diurnal animals, so its suppression in nocturnal animals related to sleep deprivation may cause different downstream effects.

With this in mind, different studies on nocturnal animal models have pointed out the obesogenic effect of light at night, precisely mediated by melatonin suppression and its deleterious effects on microbiota composition (Table 3). Namely, this disruptive environmental condition has been demonstrated to increase weight, insulin resistance and lipid influx and to produce gut microbiota dysbiosis in mice, specifically by inducing a reduction in the richness of *Blautia*, *Ruminiclostridium*, *Lachnospiraceae*, *Lactobacillus*, *Eubacterium*, *Roseburia* and *Bacteroides* (all negatively correlated with obesity). Melatonin supplementation was effective in improving circadian rhythm homeostasis and reverting these relative abundances while decreasing the abundance of *Anaerotruncus*, *Alloprevotella* and *Faecalibaculum* [11], which are related to obesity in mice [1,11]. Other authors have investigated the effect of different photoperiods on gut microbiota, finding a protective effect of 16L:8D (16 h of light, 8 h of darkness), probably mediated by the appropriate melatonin secretion and enhanced expression of melatonin receptors in laying ducks [109]. 

Similar results were found in jet-lag-induced mice, with an increase in lipid uptake and fat accumulation in the white adipose tissue as well as in the presence of *Escherichia coli* and LPS, concomitant with a reduction in angiopoietin-like 4. Oral melatonin supplementation reversed these phenotypes, probably through gut microbiota, since the treatment was not successful in microbiota-depleted animals [110].

Seasonal changes in photoperiod seem to be also related to seasonal changes in microbiota composition. In order to explore the factors involved in this apparent relationship, Shor and colleagues [111] divided pinealectomised and intact hamsters into long- or short-photoperiod groups. They found that pinealectomised animals placed on a short-day photoperiod had significantly more *Prevotella* (associated with improved glucose tolerance [112], decreased adiposity [113] and inflammatory responses [114,115]) and *Clostridium* and a lower abundance of *Desulfovibrio* as compared to animals with an intact pineal gland. The genus *Hungatella* (related to carbohydrate metabolism and energy harvest) [116], however, was enriched in pinealectomised animals under long-day photoperiods. Although these authors attribute these effects to melatonin from the pineal gland, considering the possible interplay between both local gut melatonin and pineal melatonin, indirect regulation of the former cannot be ruled out.

Feeding can also be a powerful circadian cue, especially for peripheral oscillators. In this sense, Wang et al. (2021) recently explored the effects on microbiota of restricting feeding in piglets (diurnal animals) to day or night. Although a causal relationship between melatonin and microbiota effects cannot be established, the authors found reduced concentrations of melatonin in the night-time-restricted feeding group, as well as an alteration in the diurnal rhythm and composition of the gut microbiota, with increased log ratios of *Catenibacterium:Butyrivibrio* and *Streptococcus*:*Butyrivibrio* [117], demonstrating that nocturnal fasting has a potentially protective effect on diurnal animals.

**Table 3:** Circadian cues and their effects on microbiota.

Study	Circadian Cue	Animal Model	Effects on Gut Microbiota	Other Effects	Effects of Melatonin Treatment
Hong et al., 2020 [11]	Constant light	Mouse	↓ *Blautia* ↓ *Ruminiclostridium* ↓ *Lachnospiraceae* ↓ *Lactobacillus* ↓ *Eubacterium* ↓ *Roseburia* ↓ *Bacteroides*	↑ Weight ↑ Insulin resistance ↑ Lipid influx	Reversion of dysbiosis and ↓ *Anaerotruncus* ↓ *Alloprevotella* ↓ *Faecalibaculum*
Cui et al., 2022 [109]	Long/short photoperiods	Laying ducks (diurnal)	**20 Light:4 Darkness** ↓ Microbiota α-diversity **16 Light:8 Darkness** Relative abundance of: = *Actinobacteria* = *Fusobacteria* = *Proteobacteria* = *Fusobacterium* = *Clostridium_sensu_stricto_1* = *Pectobacterium*	**16 Light:8 Darkness** ↑ Acetate ↑ Propionate, ↑ Butyrate and ↑ Total SCFA (Ileal chyme) ↓ Melatonin with increasing photoperiods.	-
Short et al., 2020 [111]	Long/short photoperiods	Hamster	**Pinealectomised, short-day photoperiod (compared to sham-pinealectomised)** ↑ *Prevotella* ↑ *Clostridium* ↓ *Desulfovibrio* **Pinealectomised, long-day photoperiod (compared to sham-pinealectomised)** ↑ *Hungatella* (Treatments did not uniformly affect OTU abundances—see paper for details).	In the presence of the pineal gland, animals with short-day photoperiods lost more weight than those with long-day photoperiods	-
Rong et al., 2021 [110]	Induced jet lag	Mouse	↑ *Escherichia coli* (↑ LPS)	↑ Lipid uptake ↑ Fat accumulation in white adipose tissue ↓ Angiopoietin-like 4	Reversed those phenotypes through gut microbiota (only in non-microbiota-depleted animals).
Wang et al., 2021 [117]	Feeding time night-restricted	Piglets (diurnal)	**Night-restricted** ↑ Log *Catenibacterium/Butyrivibrio* *↑* Log *Streptococcus/Butyrivibrio*	**Night-restricted** ↓ Melatonin (day and night) ↓ Ghrelin ↓ Dopamine ↓ Serotonin	-

“=” symbol means appropriate balance; “↑” means increase; “↓” means reduction.

## 10. Melatonin, Gut Microbiota and Disease

### 10.1. Inflammatory Bowel Disease

Chronic and recurring intestinal inflammation is a hallmark of inflammatory bowel disease (IBD), which is a catch-all name for ulcerative colitis (UC) and Crohn’s disease (CD). In 2015, IBD was estimated to affect around 3 million individuals in the United States, and its incidence and prevalence have grown globally over the past few years [118]. Since it is a complex illness with an unknown pathogenesis, it is difficult to completely treat IBD. The intricate interaction of genetic, immunologic, microbiological and environmental variables that lead to IBD makes it challenging to treat this pathology.

It is well known, however, that the gut microbiota is very much implicated in this disease, since alterations in composition and metabolism can lead to histological and anatomical effects on the gastrointestinal epithelium. Indeed, recent studies have reported that disturbances in the microbiota–host relationship are linked to IBD [119,120]. In healthy conditions, the immune system eliminates pathogenic bacteria while tolerating habitual gut microbiota [121]. If this balance is disrupted, the host’s immune response will be triggered, initiating a diseased status [122,123]. The increase in harmful bacteria [124] and the secretion of enterotoxins in the gastrointestinal tract can damage the intestinal mucosal barrier and lead to the production of inflammatory factors, which eventually will cause intestinal inflammation and immune dysfunction [123]. Faecal microbiota therapy and its beneficial effect have confirmed the essential role of the gut microbiota in IBD [125,126]. In addition, the reduced diversity of beneficial bacteria and alterations in the microbiota composition typical of these patients have been linked to an increase in ROS production and a compromised defence system in the intestinal mucosa [46]. In this sense, several studies have demonstrated that in IBD, chronic intestinal inflammation is associated with the overproduction of ROS [127,128,129]. Thus, it is not surprising that melatonin administration could be beneficial with regard to its antioxidant properties, as indicated below.

Although colitis/IBD can be triggered by sleep deprivation (as discussed in Section 8), melatonin has been proven to alleviate abdominal pain in irritable bowel syndrome patients independently of its actions on sleep [130,131]. Whether these effects of melatonin are mediated by its actions on the gut microbiota composition or other aspects of metabolism is a quite recently opened field of research. Here, we include some recent studies in which melatonin administration has been proven to alleviate colitis symptoms while affecting microbiota composition (Table 4).

The effects of melatonin on colitis have also been explored in chemically induced models of this disease. Zhu et al. (2018) showed that melatonin was effective in improving antioxidant capability in mice with colitis induced by dextran sulphate sodium (DSS), while the gut microbiota diversity, abundance and coverage did not change with the treatment. *Firmicutes* (e.g., *Coprococcus* and *Ruminococcaceae*) presented higher levels in mice treated with melatonin, while the untreated DSS group had more *Bacteroidetes* [58]. Zhao et al. (2022) explored the effects of the indoleamine in the oxazolone-induced colitis model, finding that melatonin can counteract body weight loss, colon shortening and neutrophil infiltration by suppressing the type 2 immune response. Interestingly, they suggested that these effects are mediated by the gut microbiota, whose richness and diversity at the OTU level were reduced under melatonin treatment, increasing the abundance of *Bifidobacterium*, a well-known probiotic, and reducing the abundance of different harmful bacterial genera, such as *Lachnospiraceae, Peptococcaceae* and *Desulfovibrio* [68].

Melatonin has also been administered using a nanotechnology-based treatment composed of hyaluronic acid (HA) aggregated with this indoleamine (MT) that has been demonstrated to accumulate in the inflamed colon epithelium of colitis mice, alleviating symptoms, repairing the damaged intestinal barrier and inhibiting colon inflammation. Regarding its effects on the microbiota, this conjugate (HA-MT) can restore the *Firmicutes*/*Bacteroidetes* ratio by improving the richness and diversity of the gut microbiota in mice with colitis. When analysing gut microbiota at the species level, the abundance of *Lactobacillus* increased, while that of *Bacteroides*, *Blautia* and *Streptococcus* was reduced [132].

But what is the exact mechanism by which melatonin exerts its effects on microbiota and colitis? This question has been recently addressed by Kim et al. (2020), who found that the anticolitic effects of this indoleamine require Toll-like receptor 4 (TLR4) signalling, since it alleviated induced colitis and reverted microbial dysbiosis only in wild-type but not in TLR4 knockout mice. In this experiment, melatonin significantly suppressed *Proteobacteria* (Gram-negative phylum, including *Escherichia coli* and *Salmonella*) and increased *Ruminococcaceae* family strains, which include butyrate-producing Gram-positive bacteria that are reduced in IBD faecal microbiota. These authors also demonstrated that melatonin induces Reg3b expression, an antimicrobial peptide, as well as goblet cell differentiation, through melatonin receptors and TLR4 signalling [133]. In any case, research has also demonstrated an improvement of the antioxidant capability after treatment with melatonin, so the antioxidant properties of this molecule cannot be discarded as a potential mechanism to alleviate colitis symptoms and dysbiosis.

In line with these results in animal models, descriptive studies in humans have found that melatonin concentrations are higher in faecal samples of IBD patients than in healthy control samples [134,135], and higher levels of melatonin have been negatively correlated with visceral hypersensitivity in patients with diarrhoea. Butyrate produced by *Clostridium* in IBD has been suggested to be the link for this melatonin upregulation in BON-1 cells (human neuroendocrine tumour cell lines) [135].

### 10.2. Melatonin, Gut Microbiota and Metabolic Disorders

Appetite and food intake are also influenced by the host circadian system, including its inputs and outputs [136]. In this sense, different studies have demonstrated that changes in the light–dark cycle can affect microbiota composition, which in turn can significantly impact appetite, among other aspects of the host physiology [137,138]. In parallel, melatonin production and the expression of melatonin receptors, also modulated by photoperiod, are linked to appetite/food intake regulation mediated by leptin [139,140,141,142,143].

It is well recognised that dietary composition and feeding timing also play a significant role in regulating microbiota composition and metabolism [144,145]. These changes in the gut microbiota seem to affect host physiological processes in different ways, including the modulation of food intake and appetite [146]. Therefore, a specific dietary adjustment, such as the use of probiotics, has the potential to modify the microbiota composition and functionality, which may ultimately have different effects on the host’s metabolism and control over food consumption. In zebrafish, probiotic administration of *Lactobacillus rhamnosus* seems to confirm its ability to modulate not only appetite markers but also the expression of melatonin receptors, confirming the relationship between microbial metabolism, melatonin and appetite.

On the other hand, melatonin has been detected and quantified in many different foodstuffs. Good dietary sources of melatonin include cereals, seeds and nuts [147], among which we can highlight pistachio, with 226–233 µg/g [148]. A wide range of melatonin concentrations has been also detected in fermented foods, and *Saccharomyces cerevisiae* seems to have a relevant role in melatonin formation (reviewed in [149]). 

Melatonin taken orally is absorbed from the gut and metabolised in the liver [150]. Different studies have shown that plasma melatonin concentrations and its metabolite in urine, 6-sulphatoxymelatonin, increase after the intake of foods containing indoleamine [151,152,153]. There is also evidence concerning the typical melatonin effects after the ingestion of these foods, such as the increased total antioxidative capability of serum [152]. A field to explore is thus whether melatonin contained in food can reshape the gut microbiota or whether the concentrations detected in most food are enough to produce those effects and, also, whether the effect of different food components on the microbiota could affect melatonin production in the gut.

#### 10.2.1. Breastfeeding, Melatonin and Microbiota

Breastfeeding is a particularly interesting situation in terms of the effects of orally administered melatonin on gut microbiota composition also because the melatonin concentration in breast milk is not static but rather varies following a circadian pattern. The typical night-time increase in melatonin secreted by the pineal gland is transferred through the breast milk to the nursing infant [154], transforming breast milk into a potent chrononutrient [155]. Breast milk acts as the first circadian stimulation when the baby’s circadian machinery is not yet entirely functional, entraining their developing circadian rhythms. Furthermore, apart from other actions regulating antioxidant activity, inflammation and immunity [155], the melatonin contained in breast milk could play a role in shaping the gut microbiota composition, richness and variation over time, contributing to the modulation of the absorption of different molecules in the host. In addition, melatonin from breast milk influences weight gain in infants, and it has been suggested that it limits the occurrence of sudden infant death syndrome [156] and the development of metabolic dysregulation and comorbidities, [154] such as cardiovascular diseases, over the long term [155]. It is also interesting to note that different factors in breast milk can act to regulate melatonin production, suggesting that the varying breast milk elements may differentially modulate the levels of gut melatonin, with its potential local actions on microbiota [157].

Additional evidence of the benefits of melatonin during this stage of life might be the fact that melatonin supplementation (provided in drinking water at a dosage of 0.2 mg/mL for 2 weeks) has been demonstrated to be useful in alleviating weanling stress in mice, improving body weight gain and intestinal morphology through increased richness of intestinal microbiota and by shaping its composition [12].

#### 10.2.2. Obesity

The role of melatonin in regulating energy metabolism (especially through glucose and lipid metabolism regulation) has been clearly demonstrated. In terms of oxidative stress, obesity is also characterised by an imbalance between the production of ROS and the antioxidant defence systems, resulting in a condition of oxidative stress. This has been related to the promotion of insulin resistance and metabolic syndrome by disrupting the regulation of adipokines and proinflammatory cytokines. Therefore, combining therapeutic antioxidant strategies with weight loss strategies could be beneficial [158]. In this sense, melatonin, among other antioxidants, occupies a unique position due to its antioxidant (see Section 2 of this article) and anti-inflammatory properties, as well as its role as a metabolic regulator. By modulating multiple processes involved in obesity and its associated metabolic abnormalities, melatonin shows potential therapeutic value in the treatment of obesity. Considering the topic of this review, we will focus on the possible role of microbiota in mediating the effects of melatonin in obesity.

In the previous sections, we have reviewed the actions of melatonin on gut microbiota composition. Some of these modifications can contribute to alleviating different signs of metabolic disorders, such as insulin resistance, liver steatosis, weight gain and low-grade inflammation [62]. The fact that the gut microbiota shows rhythmicity under a light/dark cycle may explain why this population of microbes may act as a possible link between the circadian clock and lipid metabolism [65]. Indeed, as previously mentioned, melatonin can influence the diurnal rhythms of gut microbiota, contribute to shaping its composition and promote lipid efflux from the intestine [64,159]. Melatonin also affects energy metabolism through its interaction with microbial metabolites, particularly with SCFAs, such as butyrate. In addition, enhancing the melatonergic pathway as a result of butyrate action also demonstrates the reciprocal influence of gut bacteria and melatonin. Some effects of melatonin seem to be mediated through the alpha-7 nicotinic receptor, while both melatonin and butyrate may control obesity through the opioidergic system [159]. Melatonin has also been shown to improve lipid metabolism in high-fat diet-fed mice, probably via microbiota along a pathway that includes acetic acid production, which shows a marked correlation with the relative abundances of *Alistipes* and *Bacteroides* [64].

Regarding the possible underlying molecular mechanisms, the circadian nuclear transcription factor, interleukin-3-regulated (NFIL3), which regulates lipid absorption and export in intestinal epithelial cells and can be activated by gut microbiota, may play a role. According to previous research, orally administered melatonin reduces the amount of LPS produced by *E. coli*, which can decrease the transcriptional inhibition of angiopoietin-like 4 (ANGPTL4) in the ileum, induced by NFIL3 through toll-like receptor 4 (TLR4)/interleukin-22 (IL-22)/STAT3 signalling. All of this, in turn, reduces ileal lipid intake and decreases fat accumulation in epididymal white adipose tissue in mice exposed to jet lag and treated with melatonin [110]. 

Apart from these direct effects of melatonin on lipid metabolism through microbiota, its microbiota-mediated actions may also include an indirect effect on muscle composition and metabolism in mice, limiting skeletal muscle frailty and allowing prolonged physical performance. Indeed, the concept of the gut–muscle axis has been recently proposed based on the reciprocal relationship between muscles and microbiota composition (extensively reviewed in [160]). In this sense, the reduction in physical activity in sarcopenic individuals could be related to different microbiota compositions and metabolisms [161,162], while overly intense physical exercise may dysregulate microbiota composition, leading to inflammation and increased risk of peptic ulcers [163]. Regarding the bones, melatonin has been shown to have a beneficial effect mediated by gut microbiota (and consequent butyrate production) on induced osteolysis [164]. 

To summarise, gut microbiota-mediated actions of melatonin in terms of obesity involve, so far, the circadian system, microbiota metabolites and skeletal muscle.

#### 10.2.3. Other Metabolic Alterations

Different epidemiological studies have revealed that circadian disruption might increase the risk of metabolic disorders. In parallel, circadian disruption has been demonstrated to affect gut permeability and microbial ecology [137,165,166,167,168]. In this sense, oral melatonin supplementation seems to be able to reverse the changes in gut microbial ecology induced by circadian disruption. 

In nocturnal rodents, melatonin has been shown to have a general beneficial role in glucose homeostasis [169], although the mechanism behind this potential relationship remains unknown. In this sense, considering that obesity and lipid dysmetabolism are known risk factors for type 2 diabetes, the previously discussed microbiota-mediated effects of melatonin might be related to a possible beneficial effect of this molecule in glucose metabolism. Apart from this indirect effect, in their matched case–control study in humans, Huang and colleagues (2022) found an altered gut microbial composition in type 2 diabetes cases, together with lower serum melatonin levels. According to their results, the underlying mechanism for this relationship may involve *Bifidobacterium*- and *Coprococcus*-mediated Trp metabolites [169]. However, the situation is controversial, since both loss-of-function and gain-of-function melatonin receptor gene MTNR1B variants seem to impair insulin secretion and increase the risk of type 2 diabetes (reviewed in [170]).

### 10.3. Oxidative Stress, Gut Microbiota and Neurological Disorders: Possible Role of Melatonin

Oxidative stress exerts harmful effects primarily by causing lipid peroxidation, damaging nucleic acids and oxidising proteins, which can have consequences on various signal transduction pathways in the central nervous system. The brain is particularly vulnerable to oxidative stress due to its high oxygen demand, especially during ATP generation through the electron transport chain. Also, different regions of the brain contain high levels of iron, which further contributes to the generation of hydroxyl radicals. Additionally, the brain has a higher concentration of polyunsaturated fatty acids, which are very affected by oxidative stress, thus making it more susceptible.

Interestingly, together with oxidative stress, the gut microbiota also seems to be an important factor in the pathogenesis of neurological disorders in a bidirectional manner (reviewed in [171]). The intestinal barrier’s homeostasis is influenced by the oxidative reduction potential of the gut microbiota, which refers to its ability to acquire electrons. Additionally, the brain/central nervous system (CNS) regulates the levels of oxidative stress in the gut through the vagal cholinergic anti-inflammatory pathway [172,173,174].

The complex interactions between gut microbiota and the host can influence the oxidative state of the CNS through the production of metabolites or the modulation of other neurotransmitters or molecules, such as melatonin. These substances can reach the CNS through systemic circulation or the vagus nerve, triggering microglia activation and neuroinflammation, increasing the production of ROS and also impacting the antioxidant systems, both directly and indirectly [173,175]. 

Notably, oxidative stress plays a role in the pathology of various chronic brain disorders, such as Alzheimer’s or Parkinson’s diseases, multiple sclerosis and depression, among others. Although speculative, these mechanisms support the hypothesis that the gut microbiota plays a role in regulating the brain’s oxidative state. In this context, based on melatonin’s antioxidant actions, this might be a molecule of interest in the study of these pathologies, considering both prevention and treatment.

**Figure 4:**
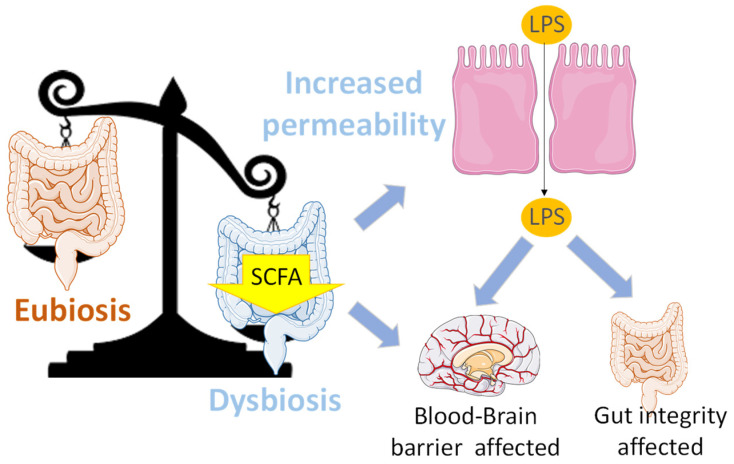
Effects of gut dysbiosis (blue gut) on gut permeability and blood–brain barrier integrity. SCFAs: short-chain fatty acids; LPS: lypopolysaccharide. This figure was created using SMART resources (Servier Medical Art), licensed under a Creative Common Attribution 3.0 Generic License. See http://smart.servier.com/ (accessed on 1 September 2023).

On the other hand, Gerwyn et al. recently suggested a relationship between the permeability of the brain–blood barrier (BBB) and the gut [176]. First, a reduction in SCFA producers increases intestinal permeability and inflammation by changing the distribution of junction proteins and increasing the transfer of LPS, which, in turn, affects the BBB and gut integrity. It should also be noted that SCFAs show anti-inflammatory action by decreasing the activity of macrophages, dendritic cells and T-lymphocytes. SCFAs are also known to be essential for the formation and maintenance of the BBB, since they modulate different pathways involved in the gut–brain axis (Figure 4).

In this section, we will review different neurological disorders in terms of their relationship with melatonin and the gut microbiota.

#### 10.3.1. Migraines

Migraines, microbiota and melatonin (the “3M” tripod) are intimately intricated [177]. Anderson [178] recently suggested that the butyrate reduction observed in dysbiosis could be related to a reduction in the availability of melatonin, with the consequent relative increase in the N-acetylserotonin/melatonin ratio. This could have consequences for the heightened glutamatergic excitatory transmission in migraines. The suboptimal functioning of the mitochondria and metabolism could produce alterations in satellite glial cells and astrocytes, which would reinforce the changes in vasoregulation and nociception observed in migraines.

#### 10.3.2. Multiple Sclerosis

Gut dysbiosis has been also proposed as a possible cause of multiple sclerosis (MS), with melatonin also being involved. As previously reviewed, gut dysbiosis has been demonstrated to lower the production of butyrate, which is a significant positive regulator of mitochondrial function and also acts by suppressing the levels and effects of ceramide, considered an important driver of multiple sclerosis pathophysiology via its effects on glial mitochondrial function and melatonin and orexin production processes. Indeed, altered regulation of the local melatonergic pathway has been suggested as an important factor for the pathophysiology of MS. According to some data, the pathophysiology of MS might be supported by changes in ceramide and mitochondrial function, particularly in glial and immune cells [179].

Melatonin also increases antimicrobial peptides, especially Reg3β, which could be useful in controlling the microbiota composition. Indeed, melatonin could exert a beneficial effect on people suffering from MS, thus presenting as a promising candidate for the treatment of this disease [180]. Also, when demyelination occurs, it has been suggested that neurons are susceptible to ROS/RNS effects [181], so, in this case, the connection microbiota–oxidative stress–melatonin might also play a role.

#### 10.3.3. Myalgic Encephalomyelitis/Chronic Fatigue Syndrome (ME/CFS)

The interplay between microbiota composition and melatonin may also play a role in the pathophysiology of myalgic encephalomyelitis/chronic fatigue syndrome, in which dysbiosis has been described [182]. As suggested and extensively reviewed by Anderson & Males [183], decreased butyrate produced by dysbiosis would attenuate the rapid epigenetic upregulation of the μ-opioid receptor, which would impact the mitochondria and immune/glial cells. This would also produce circadian effects via the β-endorphin induced by pineal melatonin, with all of this potentially being relevant to mood and cognitive deficits. Reduced levels of μ-opioid receptors and ligands may also contribute to changes in amygdala and prefrontal cortex activity that are evident in this pathology [184], as well as to emergent depression and suppressed cognition. It is precisely these effects of dysbiosis in relation to melatonin that have also been proposed to play a role in the aetiology of borderline personality disorder. In this case, the reduction in melatonin synthesis also involves the loss of the inhibition of the oestrogen receptor alpha (ERα), potentially contributing to the dysregulating effects of cyclical oestrogen in this pathology [185].

#### 10.3.4. Autism Spectrum Disorder

The human gut microbiota is currently considered to be an important factor in the development of autism spectrum disorder (ASD) in children. Although the authors clarify that it would not necessarily involve differences in the number of enzymes, the bacterial neurometabolic signature recently studied in ASD children has shown that the gut microbiota composition in these children presents decreased contents of bacterial genes for enzymes involved in the metabolism of compounds with neuroactive properties, such as melatonin [186]. Differences in mitochondrial function have been detected in autistic children compared to controls, which could be a cause of the observed elevated oxidative stress in these children (reviewed in [187]). In this regard, the antioxidant properties of melatonin potentially position it as a molecule involved in this pathology. Also, children with ASD and sleep disorder show decreased levels of melatonin and lower excretion rates of melatonin metabolites in urine [188,189,190,191], as well as reduced proportions of *Faecalibacterium* and *Agathobacter*, both butyrate producers. Melatonin levels have been found to be positively correlated with both genera [192]. Children with ASD have also shown a reduced abundance of both *Bifidobacteria* and *Prevotella*, which could reduce folate production [193,194,195]. Alterations in the gut microbiota also contribute to altered tryptophan metabolism, yielding increased levels of indolyl 3-acetic acid and indolyl lactate and the increased transformation of tryptophan into xanthurenic acid and quinolinic acid (catabolites of the kynurenine pathway) at the expense of kynurenic acid and, especially, melatonin [196].

Aside from the possible alterations in microbiota composition, some acetylserotonin O-methyltransferase (ASMT) variations have been associated with ASD [197,198,199]. These mutations significantly decrease the activity of the enzyme ASMT [199] (for details on the melatonin synthesis pathway; see Figure 2). This indicates that a weakened melatonin metabolism could be involved in impaired sleep in ASD [189]. It is not surprising then that individuals with ASD present significantly more impaired circadian rhythms [200,201] and sleep parameters, including sleep onset latency and sleep duration and efficiency [202,203]. Consistent with this, randomised double-blind controlled trials have shown a statistically significant benefit of melatonin for sleep in subjects with ASD as compared to a placebo [204,205,206]. 

Good sleep hygiene and a healthy bedtime routine instilled by behavioural training from the parents have yielded good results in improving sleep in children with ASD [207,208]. Among pharmacological treatments, melatonin shows good evidence of being effective [200,201,208,209], particularly when combined with cognitive-behavioural therapy [209]. All of these findings led to the development of a recently published hypothesis [210], which states that melatonin, circadian system functioning and microbiota are relevant factors contributing to the aetiology of ASD.

#### 10.3.5. Bipolar Disorder

Melatonin and its effect on the microbiota have also been associated with bipolar disorder due to the seasonal pattern this disorder presents [211]. Studies on this association are currently ongoing [212].

#### 10.3.6. Alzheimer’s and Parkinson’s Diseases

The role of oxidative stress in the pathogenesis of neurodegenerative diseases such as Alzheimer’s has been gaining attention in recent years [213,214,215,216,217]. In particular, the relationship between oxidative stress and the gut microbiota has attracted the attention of scientists as a possible factor in the pathoaetiology of these neurodegenerative disorders. In this regard, individuals suffering from Alzheimer’s disease present decreased populations of commensal bacteria, such as *Bifidobacterium* and *Firmicutes*, while an increased abundance of *Escherichia, Shigella* and *Bacteriodetes* has been detected, followed by increased inflammation and protein amyloid-beta accumulation [218]. The interaction between the gut microbiota, oxidative stress and the development of these diseases has been extensively reviewed in [171].

Although in the case of Alzheimer’s disease, there is no direct evidence, Zhang et al. [71] found that a mouse model with endogenous melatonin reduction, which shows microbiota dysbiosis and increased gut permeability (among other metabolic alterations), also presented Alzheimer’s disease-like phenotypes. These authors suggest that melatonin reduction may be a pathogenic factor for Alzheimer’s disease (as well as for obesity) via gut microbiota dysbiosis. Despite different studies having suggested the potential utility of therapeutic strategies aimed at reducing oxidative stress in neurodegenerative disorders [219,220,221], it is still to be conclusively demonstrated whether melatonin’s role in the pathogenesis of Alzheimer’s disease is specifically linked to its antioxidant properties. 

The scientific evidence accumulated on the effects of dysbiosis and gut permeability and melatonin on mitochondrial functioning also suggests a potential role of both aspects of physiology in the aetiology of Parkinson’s disease and may open new avenues in terms of treatment [222,223].

#### 10.3.7. Stress

Melatonin could alleviate colonic microbiota dysbiosis in mice induced by “restraint stress”, as well as the consequent intestinal inflammation, specifically by inhibiting the activation of the NF-κB pathway [224]. Also, patients with anxiety and depression have been shown to experience milder symptoms after restoring their gut microbiota [225]. 

#### 10.3.8. Chronic Pain

Recently, the possible interplay between sleep deprivation, melatonin suppression and gut dysbiosis has been reviewed in relation to chronic orofacial pain, finding several connections that might be explored [226]. Probiotics (aimed at restoring the gut microbiota composition) and melatonin supplementation have been also explored as possible therapeutic options in the management of chronic pain in fibromyalgia [227].

### 10.4. Reproductive System and Development

The beneficial effects of melatonin on the reproductive system have also been suggested to be mediated by the gut microbiota. Indeed, dietary fibre has been shown to protect against follicular atresia, at least in part by increasing melatonin and serotonin synthesis in serum and follicular fluid in a pig model [228]. Furthermore, melatonin can reduce prediabetes symptoms as well as defects in spermatogenesis while restoring normal microbiota composition and sphingosine levels altered by a rich diet in sheep, with the involvement of the gut microbiota (demonstrated via faecal transplantation) [229].

The interplay between melatonin and the gut microbiota has also been found in foetal development and placental functions in mice, with the beneficial effects of this hormone relieving barrier injury, endoplasmic reticulum stress and mitophagy through modulation of gut microbiota composition [230].

In addition, dietary melatonin supplementation is currently considered to be the first hormone associated with altering the composition (β diversity) of the bovine vaginal microbiota, leading to an increase in aerobic genera [231].

## 11. Conclusions and Future Perspectives

The multifunctional nature of melatonin makes this molecule a versatile factor in mediating different aspects of physiology and metabolism. Among these mediating functions, its multipurpose antioxidant ability should be highlighted in relation to its influence on the reciprocal interaction between the gut microbiota and other aspects of health. This influence of melatonin seems to be clear in nocturnal animal models. However, in order to confidently extrapolate results to humans, it is mandatory to also perform causal studies on diurnal animal models, especially when considering markedly circadian aspects, such as melatonin actions and sleep.

Also, human data are scarce regarding the melatonin–microbiota–health interaction, so epidemiological projects involving massive faecal and saliva/urine samplings would be desirable to correlate melatonin levels and gut microbiota composition in different populations. Human studies on probiotic effects would also be necessary to establish truly causal relationships between microbiota composition, melatonin and different aspects of health.

## Figures and Tables

**Table 2:** Sleep deprivation (SD), gut microbiota and melatonin as a possible link.

Study	Animal Model/ Duration of Sleep Deprivation (SD)	Effect of SD on the Gastrointestinal Tract	Effect of SD on Gut Microbiota	Other Effects	Melatonin Treatment (Route, Dosage, Time Period)/ Measurement	Effects of Melatonin Treatment
Gao et al., 2019 [6] and Gao et al., 2020 [5]	Mouse 72 h	Colitis	↓ *Akkermansia* ↓ *Bacteroides* ↓ *Faecalibacterium* (probiotics) ↑ *Aeromonas*	↑ Norepinephrine ↓ Plasma melatonin ↓ Antioxidant ability ↓ Anti-inflammatory cytokines ↑ Proinflammatory cytokines	20 and 40 mg/kg Intraperitoneal injections once 60 min before SD, and a single dose per day at 7:00 am for a total of 3 days	Reverse changes Improve mucosal injury and dysbiosis ↑ Plasma melatonin ↑ OTUs ↑ Diversity and richness ↑ *Bacteroidaceae* ↑ *Prevotellaceae* ↑ *Firmicutes*/*Bacteroidetes* ↑ M*oraxellaceae* ↑ *Aeromonadaceae* ↑ Anti-inflammatory cytokines ↓ Proinflammatory cytokines ↓ ROS
Wang et al., 2022 [9]	Mouse 20 h/day for 28 days	-	↑ α-diversity ↑ OTUs ↑ *Helicobacter* ↑ *Clostridium* ↓ *Bacteroidetes* ↓ *Lactobacillus*	↓ Plasma melatonin (48.91%) ↓ Antioxidant enzymes ↓ Total antioxidant capacity in intestinal tissues ↓ Anti-inflammatory cytokines (IL10, IFNγ), ↑ Glucose ↑ Norepinephrine ↑ Corticosterone ↑ Proinflammatory cytokines (IL6 and TNFα)	10^−5^ mol/L, drinking water	Reverse changes ↓ Oxidative stress ↓ Inflammatory response ↓ Dysbiosis
Gao et al., 2021 [7]	Mouse, 72 h	Mucosa injury	↓ *Faecalibacterium*	↓ Plasma melatonin ↓ Card9 expression ↓ Butyrate	20 mg/kg. Intraperitoneal injections once 60 min before SD, and a single dose per day at 7:00 am for a total of 3 days.	Reverse effects
Gao et al., 2022 [10]	Mouse 72-h No SD—effects mimicked by: *Aeromonas veronii* LPS supplementation	Mucosa injury	-	↓ Goblet cells ↓ Mucin protein ↓ Villin ↓ Tff3 mRNA ↑ TLR4 ↑ MyD 88↓ p-GSK-3β ↓ β-catenin	20 and 40 mg/kg Intraperitoneal injections once 60 min before SD, and a single dose per day at 7:00 am for a total of 3 days	Reverse effects (through MT2) of SD

**Table 4:** Effects of melatonin on microbiota composition and colitis symptoms.

Study	Animal Model	Chemical to Induce Colitis/IBD	Melatonin Treatment	Effects of Melatonin Treatment on Microbiota Composition	Effects of Melatonin Treatment on Colitis Symptoms
Zhu et al., 2018 [58]	Mouse	Dextran Sulphate Sodium (DSS)	Drinking water 0.2 mg/L	= Diversity = Abundance = Coverage ↑ *Firmicutes* ↑ *Bacteroidetes* (with no melatonin)	Increased antioxidant capability
Jing et al., 2022 [132]	Mouse	Dextran Sulphate Sodium (DSS)	Melatonin + hyaluronic acid (aggregates)	Alleviate dysbiosis Restore *Firmicutes*/*Bacteroidetes* ↑ Richness ↑ Diversity ↑ Lactobacillus ↓ *Bacteroides, Blautia, Streptococcus*	Restoration of the intestinal barrier Inhibition of colon inflammation
Zhao et al., 2022 [68]	Mouse	Oxazolone	Via gavage 50 mg/kg	↓ Richness at the OTU level ↓ Diversity at the OTU level ↑ *Bifidobacterium* ↓ *Desulfovibrio* ↓ *Peptococcaceae* ↓ *Lachnospiraceae*	Counteracting body weight loss Counteracting colon shortening Neutrophil infiltration Suppression of type 2 immune response
Kim et al., 2020 [133]	Mouse (wild type and TLR4 knockout)	DSS	Oral and rectal 10 mg/kg/day	Revert dysbiosis ↑ Richness ↑ Diversity ↓ *Proteobacteria* ↑ *Ruminococcaceae* ↓= *Bacteroidetes* ↑= *Firmicutes*	↓ Disease activity index Alleviation of the shortening of colon and histopathologic features
